# Evaluating ovarian blood supply anatomy and variability via digital subtraction angiography in patients with cesarean scar pregnancy undergoing uterine artery embolization

**DOI:** 10.3389/fmed.2026.1730033

**Published:** 2026-03-26

**Authors:** Guo Zheng, Huiling Gao, Zhao Jin, Yue Wen, Hongzhen Xu, Shuyuan Dong, Shuang Li

**Affiliations:** Department of Interventional Radiology, Heibei Maternity Hospital, Shijiazhuang, China

**Keywords:** blood supply, digital subtraction angiography, ovarian artery, uterine artery, uterine artery embolization

## Abstract

**Background:**

Preservation of ovarian perfusion is a critical consideration during uterine artery embolization (UAE), as inadvertent disruption can reduce ovarian reserve. Given the complex and variable anatomy of ovarian blood supply, this study aimed to characterize its anatomical origins and variations using digital subtraction angiography (DSA) in patients undergoing UAE for cesarean scar pregnancy (CSP).

**Methods:**

This retrospective study included patients with type II or III CSP who underwent first-time UAE at Heibei Maternity Hospital, China, between August 2020 and October 2024. Pre-embolization DSA, including abdominal aortography and selective uterine arteriography, was analyzed to assess visualization of left and right ovarian arteries, uterine artery ovarian branches, and ovarian parenchymal staining. Statistical comparisons between bilateral vessels were performed using chi-square tests.

**Results:**

Among 243 patients, the left ovarian artery was visualized in 42.8% and the right in 44.86%. Bilateral ovarian arteries were visualized in 18.52%, and neither artery was visualized in 20.58%. Uterine artery ovarian branches were visualized in 39.51% (*n* = 96, left) and 38.27% (*n* = 93, right), with parenchymal staining observed in 70.83% (*n* = 68) and 67.74% (*n* = 63) of these, respectively. No statistically significant differences were found between sides for ovarian arteries or uterine branches (*P* > 0.05). Complete visualization of all four potential vascular sources occurred in 8.23% of cases, while complete absence was seen in 13.58%.

**Conclusion:**

Ovarian blood supply demonstrates substantial interindividual variation, with uterine artery ovarian branches contributing significantly to perfusion. No significant bilateral differences were identified, indicating that variability arises at the individual rather than side-specific level. Comprehensive bilateral vascular assessment with DSA remains essential to guide embolization strategies that minimize the risk of ovarian dysfunction.

## Introduction

Uterine artery embolisation (UAE) is a well-established, minimally invasive intervention for managing various causes of gynecological hemorrhage, including symptomatic uterine fibroids and postpartum hemorrhage (1). In recent years, its application has also been extended to the management of another high-risk gynecological condition associated with significant hemorrhage risk—cesarean scar pregnancy (CSP). CSP represents a rare but increasingly recognized form of ectopic pregnancy in which the gestational sac implants at the site of a previous cesarean section scar. CSP carries a high risk of severe hemorrhage and uterine rupture if not managed appropriately. UAE has been widely employed as a fertility-preserving strategy in CSP, effectively controlling hemorrhage and facilitating subsequent surgical or medical management. While the UAE is generally effective, preservation of ovarian blood supply during the procedure to prevent subsequent ovarian dysfunction remains a significant clinical challenge (1–3). This challenge is compounded by the considerable anatomical variability of ovarian vascular supply between individuals, highlighting the importance of precise preoperative evaluation (2, 4, 5).

Digital subtraction angiography (DSA) provides detailed visualization of the pelvic vasculature and allows identification of the origins of ovarian arteries before embolization (4, 6, 7). Understanding these variations is critical, as inadvertent compromise of ovarian perfusion during UAE can lead to decreased ovarian reserve and early ovarian failure, particularly in women over 40 years (1, 4, 7). Despite this, the relationship between angiographic findings and ovarian characteristics is still not fully understood, and few studies systematically describe ovarian artery anatomy as seen on DSA (2, 5, 8). Therefore, this study aimed to characterize its anatomical origins and variations using DSA in patients undergoing UAE for CSP.

## Materials and methods

### Study design and participants

This retrospective observational study enrolled patients with CSP who underwent UAE at Heibei Maternity Hospital between August 2020 and October 2024. Inclusion criteria: (1): Confirmed diagnosis of type II or III CSP; (2): Underwent first-time UAE treatment; (3): No history of infertility in either partner. Exclusion criteria: (1): Psychiatric disorders; (2): Severe organ dysfunction; (3): Factors (anti-Müllerian hormone (AMH) < 1.1 ng/ml, or follicle-stimulating hormone (FSH) > 20 IU/L.) affecting natural conception; (4): Refusal to undergo UAE; (5): Ovarian artery malformations; (6): Contraindications to DSA. The study was approved by the Institutional Review Board of Heibei Maternity Hospital (Approval number 20220024). Given the retrospective design of the study, the requirement for individual informed consent was waived by the Institutional Review Board.

### Data collection and definition

**FIGURE 1:**
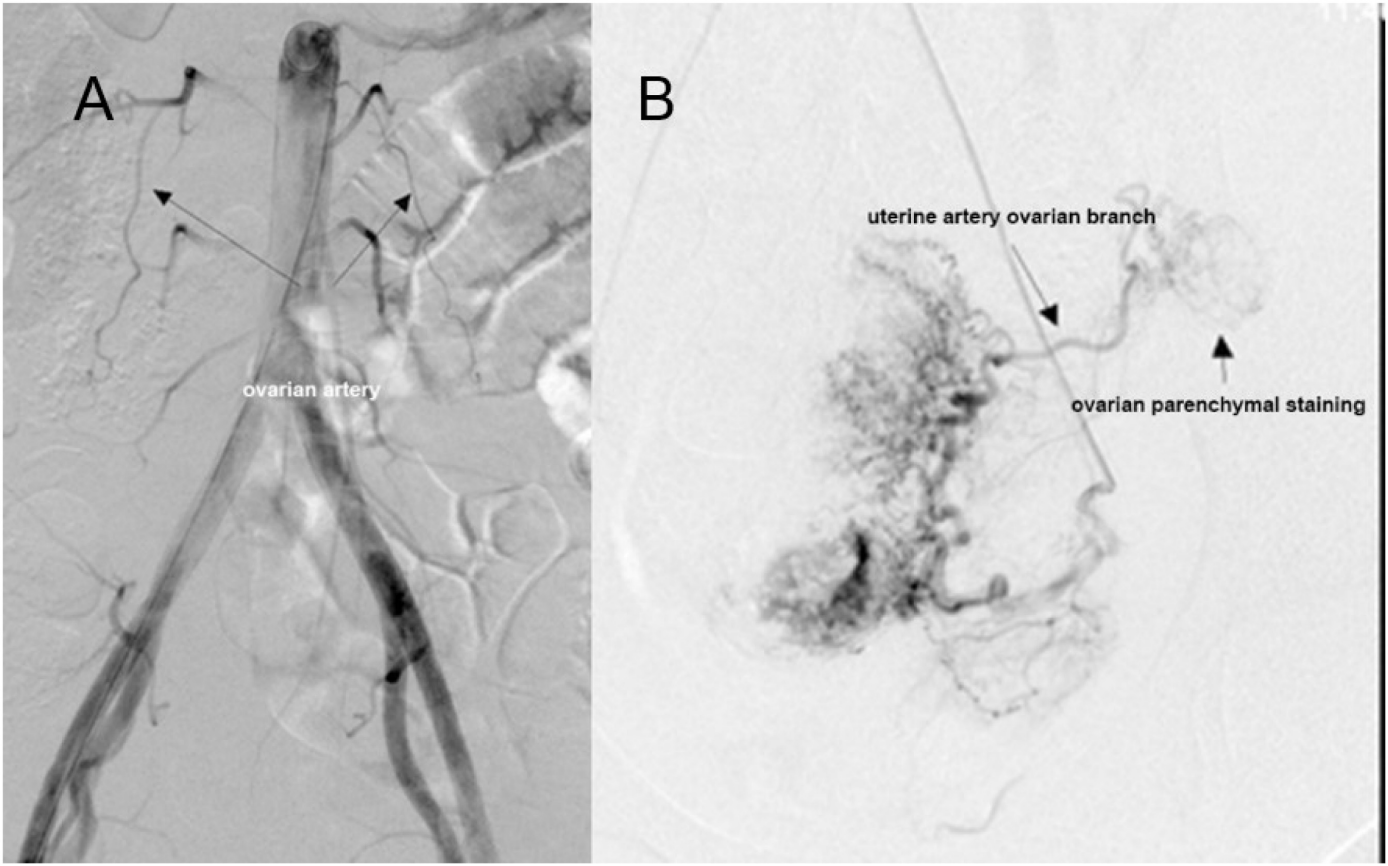
Abdominal aorta-uterine artery angiography. **(A)** Angiography of the upper segment of the abdominal aorta shows the bilateral ovarian arteries arising from the abdominal aorta. **(B)** Uterine artery angiography demonstrates the ovarian branch of the uterine artery and ovarian vascular staining.

Patient clinical data were systematically collected from medical records, including relevant clinical history. Angiographic characteristics were also recorded, encompassing visualization status of left and right ovarian arteries, ovarian branches from the left and right uterine arteries, and ovarian parenchymal staining (Figure 1). Digital subtraction angiography (DSA) images were independently reviewed by two attending physicians with a senior title or above. Any disagreements in image interpretation were resolved by a chief physician.

All procedures were performed via femoral artery access using a 5F introducer sheath. A 5F pigtail catheter was advanced under fluoroscopic guidance to the inferior border of the first lumbar vertebra’s inferior border within the abdominal aorta for initial angiography to visualize bilateral ovarian arteries. The pigtail catheter was then exchanged for a 5F uterine artery catheter, which was selectively advanced into each uterine artery. Repeat angiography was conducted to evaluate the visualization of ovarian branches originating from the ascending uterine artery and the presence of ovarian parenchymal contrast staining. No microcatheters were used. Angiographic parameters were as follows: abdominal aortography utilized 12–15 mL of contrast medium at 10–12 mL/s, while uterine arteriography used 3–5 mL at 2–3 mL/s.

Ovarian parenchymal staining was defined as late-phase capillary blush within the ovarian tissue on digital subtraction angiography, characterized by diffuse, homogeneous contrast opacification of the ovarian parenchyma during the venous phase (3–5 s after arterial phase), indicating actual tissue perfusion rather than transient arterial flow.

### Statistical analysis

Data were analyzed using SPSS software, version 26.0 (IBM Corp., Armonk, NY, United States). Categorical variables were expressed as numbers and percentages, whereas continuous variables were expressed as mean ± standard deviation (SD). Comparisons of visualization rates between bilateral ovarian arteries or uterine artery branches were performed using chi-square tests. Two-sided *P*-values *P* < 0.05 were considered statistically significant.

## Results

### Basic characteristics

**FIGURE 2:**
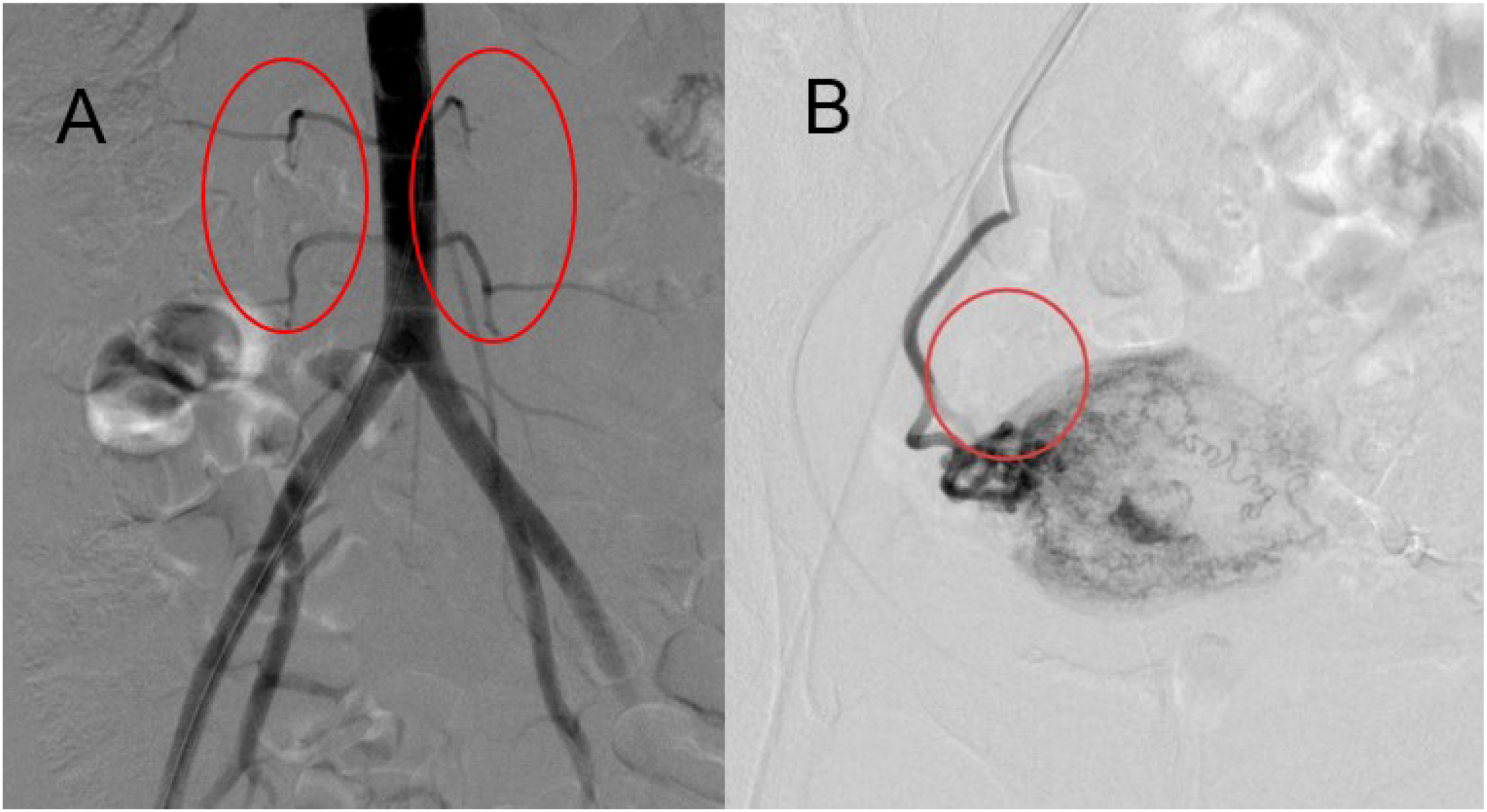
Negative findings of ovarian arteries and ovarian branches on abdominal aorta-uterine artery angiography. **(A)** No visualization of bilateral ovarian arteries on upper abdominal aorta angiography. **(B)** No ovarian branches or ovarian vascular staining in uterine ascending branch supply region on uterine artery angiography.

A total of 243 patients were included in the study. The left ovarian artery was visualized in 104 cases (42.80%) and not visualized in 139 cases (57.20%). The right ovarian artery was visualized in 109 cases (44.86%) and not visualized in 134 cases (55.14%). Bilateral visualization patterns showed that both ovarian arteries were simultaneously visualized in 45 cases (18.52%), whereas neither artery was visualized in 50 cases (20.58%) (Figure 2 and Table 1).

**TABLE 1:** Summary of ovarian artery and uterine artery branch visualization and parenchymal staining.

Variables	Description	Number (*n*)	Percentage (%)
Abdominal aortography			
Left ovarian artery	Visualized	104	42.80
	Non-visualized	139	57.20
Right ovarian artery	Visualized	109	44.86
	Non-visualized	134	55.14
Bilateral ovarian arteries	Both visualized	45	18.52
	Neither visualized	50	20.58
Uterine arteriography			
Left uterine artery ovarian branch	Visualized	96	39.51
	Visualized with parenchymal staining	68	27.98 (68/96, 70.83% of visualized)
	Non-visualized	147	60.49
Right uterine artery ovarian branch	Visualized	93	38.27
	Visualized with parenchymal staining	63	25.93 (63/93, 67.74% of visualized)
	Non-visualized	150	61.73
Ovarian-related vascular visualization patterns			
Ipsilateral ovarian artery and branch	Both visualized (left)	36	14.81
	Both visualized (right)	35	14.81
Ipsilateral ovarian artery and branch	Neither visualized (left)	78	14.40
	Neither visualized (right)	80	32.92
All four sources visualized		20	8.23
All four sources neither visualized		33	13.58
≥ 1 left-sided ovarian vessel visualized		165	67.90
Left ovarian artery visualized (primary source)		104	42.80
≥ 1 right-sided ovarian vessel visualized		163	67.08
Right ovarian artery visualized (primary source)		109	44.86

The left uterine artery ovarian branch was visualized in 96 cases (39.51%), with ovarian parenchymal staining present in 68 of these cases [70.83% of visualized branches (*n* = 96)]. The branch was not visualized in 147 cases (60.49%). Similarly, the right uterine artery ovarian branch was visualized in 93 cases (38.27%), with parenchymal staining observed in 63 cases [67.74% of visualized branches (*n* = 93)], and non-visualization occurred in 150 cases (61.73%) (Table 1).

**FIGURE 3:**
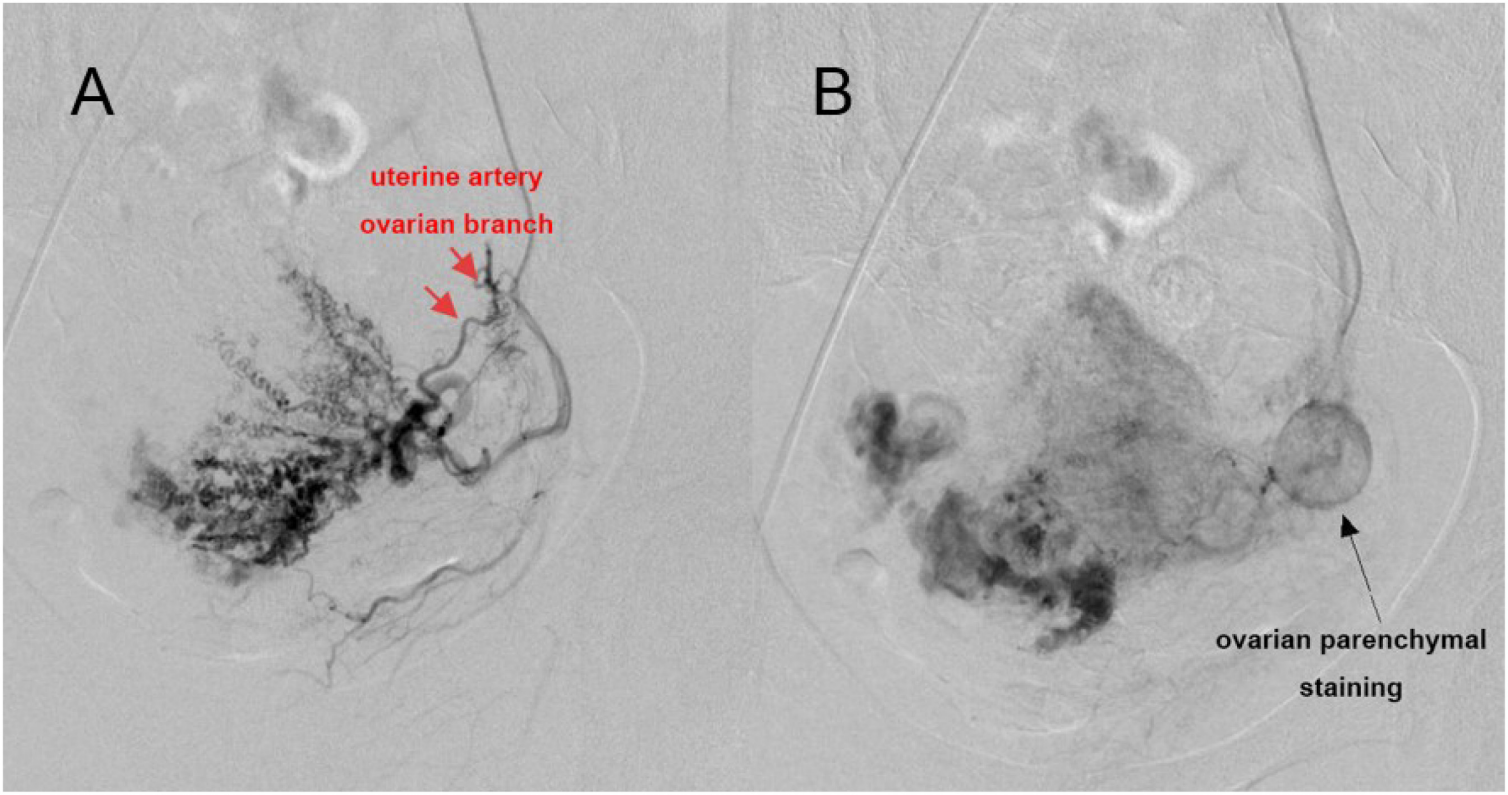
Ovarian branch and ovarian staining on uterine artery angiography. **(A)** Visualization of ovarian branch in early arterial phase on uterine artery angiography. **(B)** Round-like ovarian staining in late arterial phase on uterine artery angiography.

Concurrent visualization of the ipsilateral ovarian artery and uterine artery ovarian branch was observed in 36 left-sided cases (14.81%) and 35 right-sided cases (14.40%). Simultaneous non-visualization of both vessels occurred in 78 left-sided cases (32.10%) and 80 right-sided cases (32.92%). Complete visualization from all four potential sources (both ovarian arteries and both uterine artery ovarian branches) was achieved in 20 cases (8.23%) (Figure 3), whereas complete absence of vascular opacification from all four sources was observed in 33 cases (13.58%) (Table 1).

### Bilateral differences in ovarian blood supply

**FIGURE 4:**
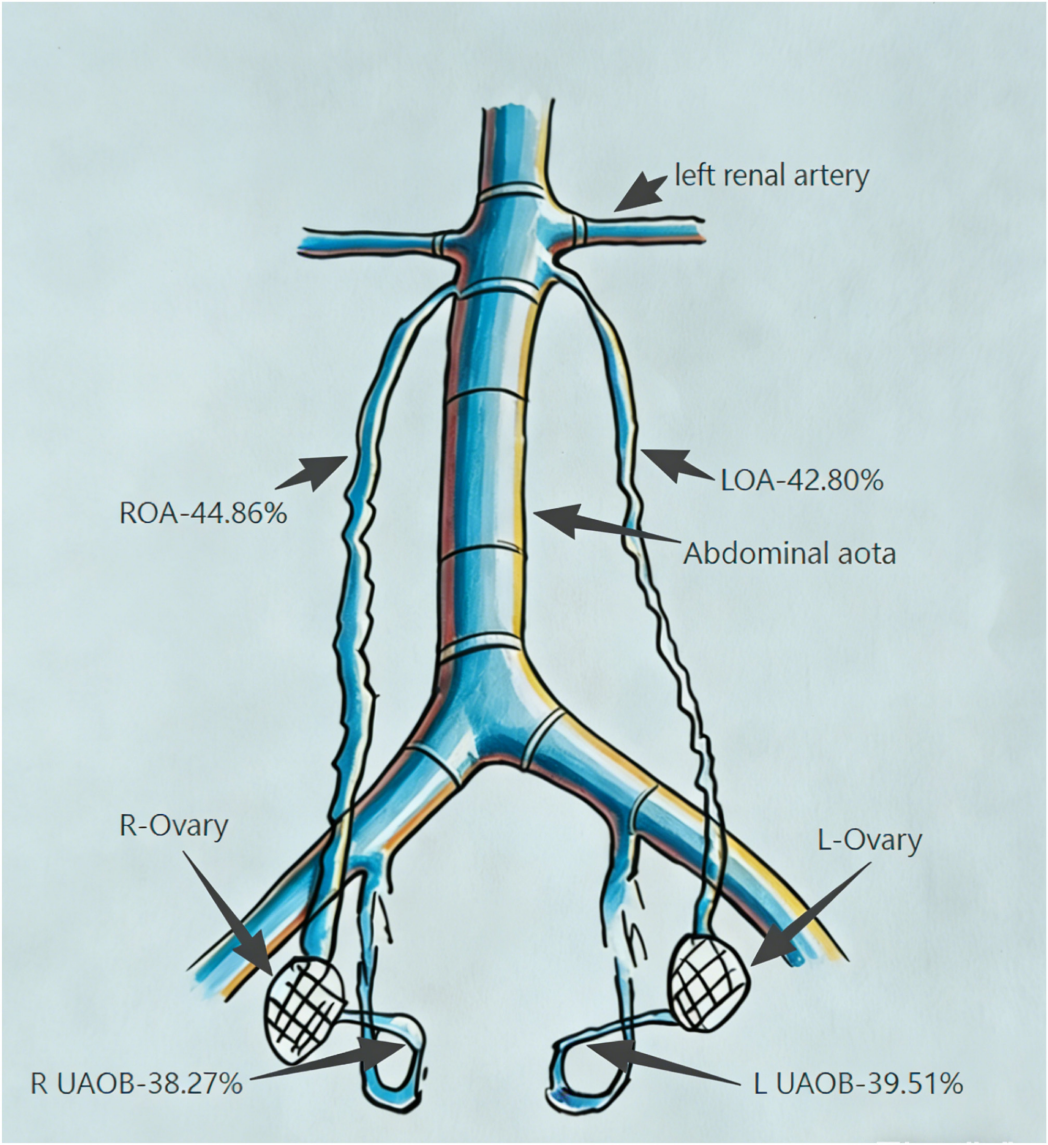
Four main vascular supply origins and their relative visualization frequencies. LOA, left ovarian artery; ROA, right ovarian artery; UAOB, uterine artery ovarian branch.

No statistically significant differences were observed in the bilateral opacification rates of ovarian arteries arising from the abdominal aorta (*P* = 0.722). Similarly, the comparison between cases with both ovarian arteries visualized (45 cases, 18.52%) and those with neither visualized (50 cases, 20.58%) revealed no significant difference (*P* = 0.608). Bilateral uterine artery ovarian branches showed comparable visualization rates (*P* = 0.782), and no significant differences were noted in ovarian parenchymal staining between the two sides (*P* = 0.635) (Tables 2, 3). Details of the four main vascular supply origins and their relative visualization frequencies are illustrated in Figure 4 and Table 4.

**TABLE 2:** Bilateral ovarian artery and uterine artery branch visualization.

Variables	Visualized	Non-visualized	*P*-value
Ovarian artery			
Left	104	139	0.722
Right	109	134	
Uterine artery ovarian branch			
Left	96	147	0.782
Right	93	150	

**TABLE 3:** Bilateral Ovarian Artery and Uterine Artery Branch Parenchymal Staining.

Variables	Staining	Non-staining	*P*-value
Uterine artery ovarian branch			0.635
Left	68	28	
Right	63	30	

**TABLE 4:** Distribution Patterns of ovarian blood supply on left and right sides.

Vascular pattern	Left side (*n* = 243)	Right side (*n* = 243)
Both ovarian artery and uterine artery ovarian branch visualized	36 (14.81%)	35 (14.40%)
Only ovarian artery visualized	68 (27.98%)	74 (30.45%)
Only uterine artery ovarian branch visualized	60 (24.69%)	58 (23.87%)
Neither ovarian artery nor uterine artery ovarian branch visualized	79 (32.51%)	76 (31.28%)

Interestingly, the logistic regression results revealed that age was associated with the visualization of both right uterine artery ovarian branch and right ovarian parenchymal staining, however, the visualization of left side organs was not associated with age (Supplementary Table 1).

## Discussion

This retrospective study used DSA to characterize ovarian vascular supply. No statistically significant bilateral differences were detected, indicating that ovarian vascular patterns are broadly comparable between left and right sides. Rather than demonstrating laterality-specific variation, these results highlight the overall heterogeneity of ovarian perfusion and the multiple potential vascular sources contributing to ovarian blood supply. Accordingly, a thorough bilateral assessment remains essential before UAE, not to compare left versus right dominance, but to identify individual variations that may influence ovarian preservation.

The comparable opacification rates between left and right ovarian arteries, along with substantial rates of bilateral non-visualization, reflect the complexity and heterogeneity of ovarian blood supply among individuals. This variability underscores that ovarian perfusion is not solely dependent on a single arterial source but rather involves a dynamic interplay between primary and collateral vessels. Approximately one-fifth of patients relied on alternative sources, such as uterine artery branches or collateral vessels, for ovarian perfusion. These findings extend previous reports by Smith and Johnson (8) and Brown and Patel, providing additional quantitative evidence for the prevalence of compensatory perfusion pathways and highlighting the critical role of pre-procedural DSA in identifying patients at risk of inadvertent ovarian ischemia during UAE.

Uterine artery ovarian branches were visualized in 38–39% of cases, with a high proportion demonstrating ovarian parenchymal staining. The relatively low simultaneous visualization of the ipsilateral ovarian artery and uterine artery branch (14.4–14.81%) suggests the presence of compensatory perfusion mechanisms, which may be adaptive responses to variable arterial anatomy. Pre-UAE assessment of these branches is therefore crucial, not only to preserve alternative perfusion routes but also to inform individualized embolization techniques that minimize the risk of iatrogenic ovarian injury. In the present study, although no significant difference was found between bilateral branches, the individual variability should be noticed. DSA could provide accurate, high-resolution vasculature (9), combined with a deep learning vessel segmentation tool (10). Future studies could extract personalized vasculature and help design the UAE protocol.

Chi-square analysis revealed no significant differences between left and right ovarian arteries or uterine branches, however, the regression analysis showed that visualization of the left side uterine artery ovarian branch was associated with age. These findings indicated that age was a potential affecting factor for vessel examination in clinical scenarios (11). Another interesting finding was 13.58% of patients exhibited no detectable ovarian vascular supply from any of the four assessed sources, implying a reliance on less prominent collateral vessels, such as the mesenteric or lumbar arteries, however, to our knowledge, no study has focused on the prevalence of collateral vessels, and the findings of our study may serve as a pilot results. This finding emphasizes that unilateral angiographic assessment cannot reliably predict contralateral or overall ovarian perfusion, reinforcing the need for comprehensive bilateral evaluation. These findings emphasize that unilateral assessment cannot reliably predict contralateral perfusion, supporting individualized intraoperative embolization strategies (12), and may help guide risk stratification for ovarian dysfunction post-UAE. Except for the DSA technique, the magnetic resonance (MR) could also help the assessment of uterine and ovarian arteries prior UAE Mori et al. showed that 97% uterine artery origins were demonstrated clearly at MR angiography (13), and this non-invasive technique could be used as a follow-up examination tool to evaluate the long-term effect of UAE.

Although this study did not quantitatively assess post-embolization ovarian function or long-term fertility outcomes, the observed vascular patterns raise important clinical questions regarding ovarian preservation. Variations in ovarian blood supply visualization—particularly the absence of both ovarian artery and uterine artery branch opacification (“dual non-visualization”)—may indicate a predominant reliance on collateral or accessory vessels (e.g., mesenteric or lumbar arteries) for ovarian perfusion. In such cases, the UAE might inadvertently compromise these alternative pathways, potentially increasing the risk of ovarian ischemia and subsequent decline in ovarian reserve. Conversely, patients with robust visualization of both ovarian artery and uterine artery branch (“dual visualization”) might possess redundant perfusion routes, potentially offering greater resilience against iatrogenic vascular disruption.

Previous studies have suggested that inadvertent embolization of ovarian arteries or their significant branches during UAE is associated with transient or permanent reductions in anti-Müllerian hormone (AMH) levels and antral follicle counts (AFC), markers of ovarian reserve. For instance, Kaump et al. reported that non-target embolization of ovarian vessels could lead to accelerated ovarian aging, particularly in women over 40 (1). Similarly, Lee et al. emphasized that the presence of functional uterine artery ovarian branches correlates with post-UAE ovarian perfusion preservation, potentially mitigating the risk of premature ovarian insufficiency. The heterogeneity of vascular patterns observed in our cohort underscores the need for individualized embolization strategies. Patients with “dual non-visualization” patterns might benefit from more conservative embolization endpoints or enhanced imaging techniques (e.g., cone-beam CT or superselective angiography) to identify and preserve cryptic collateral vessels. Prospective studies incorporating serial assessments of AMH, AFC, and fertility outcomes are warranted to determine whether specific angiographic patterns predict ovarian functional resilience or vulnerability after UAE for CSP.

Overall, these data highlight the intricate and variable nature of ovarian vascular anatomy, the compensatory role of uterine artery branches, and the critical importance of thorough preoperative vascular assessment to optimize ovarian function preservation. Future research should include longitudinal assessments of ovarian function and explore the development of a classification system for perfusion phenotypes, particularly for extreme patterns of complete visualization or complete absence. Such data could inform personalized embolization strategies and improve outcomes for ovarian preservation.

### Limitations and future perspective

This study was limited to patients undergoing UAE for CSP, which may reduce generalizability to other populations, such as those with fibroids or abnormal uterine bleeding. Post-procedural ovarian function was not evaluated, so the functional significance of vascular patterns remains to be validated. For future prospective study design, we would incorporate not only the ovarian function tests, but also the functional imaging results to better characterize ovarian function. The study population will also be extended to include patients with various diseases. And we hope our study could provide insights for the female population.

## Conclusion

This study provides quantitative evidence of ovarian vascular heterogeneity and the significant contribution of uterine artery ovarian branches to ovarian perfusion. As no significant bilateral differences were observed, the findings underscore that ovarian perfusion patterns are variable at the individual level rather than side-specific. Future studies should focus on functional validation of these perfusion patterns and their implications for personalized ovarian preservation strategies.

## Data Availability

The original contributions presented in this study are included in this article/supplementary material, further inquiries can be directed to the corresponding author.

## References

[1] Kaump GR Spies JB. The impact of uterine artery embolization on ovarian function. *J Vasc Interv Radiol.* (2013) 24:459–67. 10.1016/j.jvir.2012.12.002 23384832

[2] Ozen M Momin S Myers CB Hoffman M Raissi D. Primary bilateral ovarian artery embolization for uterine leiomyomatosis in the setting of a rare anatomic variant - hypoplastic uterine arteries. *Radiol Case Rep.* (2021) 16:2426–8. 10.1016/j.radcr.2021.05.069 34257773 PMC8260744

[3] Donnez J Dolmans MM. Uterine fibroid management: from the present to the future. *Hum Reprod Update.* (2016) 22:665–86. 10.1093/humupd/dmw023 27466209 PMC5853598

[4] Pelage JP Walker WJ Le Dref O Rymer R. Ovarian artery: angiographic appearance, embolization and relevance to uterine fibroid embolization. *Cardiovasc Intervent Radiol.* (2003) 26:227–33. 10.1007/s00270-002-1875-3 14562969

[5] Thomas RP Bastian MB Viniol S König AM Amin SS Eldergash O et al. Digital variance angiography in selective lower limb interventions. *J Vasc Interv Radiol.* (2022) 33:104–12. 10.1016/j.jvir.2021.09.024 34653607 PMC8844582

[6] Kröncke T. An update on uterine artery embolization for uterine leiomyomata and adenomyosis of the uterus. *Br J Radiol.* (2023) 96:20220121. 10.1259/bjr.20220121 36222200 PMC9975358

[7] Dariushnia SR Nikolic B Stokes LS Spies JB. Quality improvement guidelines for uterine artery embolization for symptomatic leiomyomata. *J Vasc Interv Radiol.* (2014) 25:1737–47. 10.1016/j.jvir.2014.08.029 25442136

[8] Cappelli A Mosconi C Cocozza MA Brandi N Bartalena L Modestino F et al. Uterine artery embolization for the treatment of symptomatic uterine fibroids of different sizes: a single center experience. *J Pers Med.* (2023) 13:906. 10.3390/jpm13060906 37373895 PMC10302260

[9] Leonhardt H Thilander-Klang A Båth J Johannesson M Kvarnström N Dahm-Kähler P et al. Imaging evaluation of uterine arteries in potential living donors for uterus transplantation: a comparative study of MRA, CTA, and DSA. *Eur Radiol.* (2022) 32:2360–71. 10.1007/s00330-021-08350-6 34767069 PMC8921132

[10] Sasaki S Kitaguchi D Noda T Matsuzaki H Hasegawa H Takeshita N et al. Deep learning-based vessel and nerve recognition model for lateral lymph node dissection: a retrospective feasibility study. *Langenbecks Arch Surg.* (2025) 410:310. 10.1007/s00423-025-03882-7 41144024 PMC12559106

[11] Wooldridge AL Pasha M Chitrakar P Kirschenman R Quon A Spaans F et al. Advanced maternal age impairs uterine artery adaptations to pregnancy in rats. *Int J Mol Sci.* (2022) 23:9191. 10.3390/ijms23169191 36012456 PMC9409016

[12] Wang MQ Liu FY Duan F Wang ZJ Song P Song L. Ovarian artery embolization supplementing hypogastric-uterine artery embolization for control of severe postpartum hemorrhage: report of eight cases. *J Vasc Interv Radiol.* (2009) 20:971–6. 10.1016/j.jvir.2009.04.049 19555891

[13] Mori K Saida T Shibuya Y Takahashi N Shiigai M Osada K et al. Assessment of uterine and ovarian arteries before uterine artery embolization: advantages conferred by unenhanced MR angiography. *Radiology.* (2010) 255:467–75. 10.1148/radiol.10091339 20332375

