# Supplementary material for: Evaluating ovarian blood supply anatomy and variability via digital subtraction angiography in patients with cesarean scar pregnancy undergoing uterine artery embolization
Source: Front Med (Lausanne). 2026 Mar 26;13:1730033. doi: 10.3389/fmed.2026.1730033 (PMC13062180; doi:10.3389/fmed.2026.1730033)
Supplement: Supplementary file 1 [file Table_1.docx]

**Supplementary Table 1**. Logistic regression analysis of visualization results.

|  | **Variables** | **OR (95% CI)** | **P value** |
| --- | --- | --- | --- |
| Right ovarian artery | age | 2.766 (0.956-1.083) | 0.5855 |
|  | number of term pregnancies | 3.889 (0.997-2.029) | 0.0934 |
|  | preterm birth | 2.718 (0.373-2.644) | 0.9997 |
|  | number of abortions | 2.296 (0.678-1.009) | 0.0665 |
| Right uterine artery ovarian branch | age | 2.513 (0.862-0.983) | **0.0146** |
|  | number of term pregnancies | 1.990 (0.434-0.988) | 0.0840 |
|  | preterm birth | 5.851 (0.664-4.951) | 0.2604 |
|  | number of abortions | 2.951 (0.887-1.324) | 0.4378 |
| Right ovarian parenchymal staining | age | 2.492 (0.845-0.984) | **0.0189** |
|  | number of term pregnancies | 2.185 (0.463-1.132) | 0.2907 |
|  | preterm birth | 3.881 (0.442-3.746) | 0.5645 |
|  | number of abortions | 2.875 (0.841-1.317) | 0.6294 |
| Left ovarian artery | age | 2.654 (0.918-1.037) | 0.4370 |
|  | number of term pregnancies | 2.585 (0.712-1.241) | 0.7016 |
|  | preterm birth | 2.061 (0.266-1.851) | 0.5018 |
|  | number of abortions | 2.812 (0.855-1.254) | 0.7309 |
| Left uterine artery ovarian branch | age | 2.781 (0.962-1.088) | 0.4710 |
|  | number of term pregnancies | 2.902 (0.817-1.443) | 0.6460 |
|  | preterm birth | 2.571 (0.364-2.469) | 0.9039 |
|  | number of abortions | 2.674 (0.812-1.191) | 0.8652 |
| Left ovarian parenchymal staining | age | 2.863 (0.986-1.124) | 0.1304 |
|  | number of term pregnancies | 2.532 (0.652-1.226) | 0.6319 |
|  | preterm birth | 2.374 (0.279-2.294) | 0.7804 |
|  | number of abortions | 2.836 (0.849-1.273) | 0.6862 |
